# Re-Analysis of Single-Nucleus Transcriptomics Reveals Diverse Dorsal Root Ganglia Macrophage Responses Following Peripheral Nerve Injury

**DOI:** 10.3390/biomedicines10123295

**Published:** 2022-12-19

**Authors:** Nea Korvenlaita, Lauri Louhivuori

**Affiliations:** Orion Corporation, Orion Pharma, 20380 Turku, Finland

**Keywords:** peripheral nerve injury, dorsal root ganglia, macrophage, neuropathic pain, single nucleus transcriptomics

## Abstract

An increasing amount of evidence points to an important role of macrophages in peripheral nerve injury (PNI) and associated pain. Peripheral nerve macrophages facilitate the regeneration, while dorsal root ganglia (DRG) macrophages might propagate the injury after a PNI. These differences might be explained by various in vivo models of PNIs or non-uniform methodologies to phenotype the macrophages. Unbiased methods to phenotype macrophages using single whole cell or nucleus transcriptomics have been rarely applied on PNIs outside the nerves themselves. Here, we compare the effects of the transection or crush of the sciatic nerve and spinal nerve transection on the DRG macrophage phenotypes utilizing a publicly available single-nucleus transcriptomic DRG dataset. Our results demonstrate that unique and time-dependent DRG macrophage gene expression profiles were produced by the three PNI models with particular macrophage clusters being enriched that were dependent on the severity of the neuronal injury score. PNI associated DRG macrophages were not purely anti- or pro-inflammatory. These results suggest that various functions of DRG macrophage subtypes are carefully orchestrated upon a PNI. These findings open a new avenue for studying the DRG macrophage subtypes in PNIs and encourage further unbiased phenotyping efforts to better understand their relevance in neuropathic pain.

## 1. Introduction

The cellular and molecular interactions between the spinal dorsal horn neurons and microglia, the resident macrophages of the central nervous system (CNS), are increasingly appreciated as playing an important role in the induction and maintenance of neuro-pathic pain following peripheral nerve injury (PNI) [1]. Concurrent to the activation of the spinal cord microglia, studies have demonstrated a significant expansion and proliferation of macrophages around the injured sensory neurons in dorsal root ganglia (DRG) ipsilateral to the site of the injury [2]. The depletion of macrophages in the DRG has been shown to prevent the development of nerve injury-induced mechanical hypersensitivity [3], suggesting that pro-nociceptive neuronal and non-neuronal cellular interactions also occur in the DRG and may contribute to and be required for neuropathic pain initiation and maintenance. DRG macrophages express both pro-inflammatory and regenerative genes in a temporal manner after a nerve injury [4], and the infiltration of the phagocytic macrophages to the site of a nerve injury is crucial for the regeneration of the nerve especially after a sciatic crush injury [5,6]. These studies suggest that while a PNI induces macrophage responses in different peripheral and central tissues, the consequences of such effects are highly dynamic, location dependent, and specific to the type of nerve injury.

Recent studies have utilized single-cell RNA sequencing to delve into the temporal and spatial localization of macrophages with unique gene expression profiles at the sites of pain modulation in both the spinal cord [7,8] and peripheral nerves [2,9]. However, the phenotypic changes of the macrophages in the DRG during a PNI remains unclear. A recent review on the use of single-cell sequencing techniques in neuropathic pain studies clearly indicates the focus on the analysis of neuronal subtypes, while the contribution of other cell types is still poorly understood at the resolution provided by single-cell sequencing [10]. As macrophages are considered to be one of the most plastic cells of the immune system, marked by their capacity to profoundly adapt to their tissue of residence and to change immensely during tissue injury [9], we hypothesized that the gene expression signatures of the macrophages in the DRG are highly likely to also display temporal changes dependent on their tissue environment under homeostasis and upon a PNI.

Here, we aim to reveal the heterogeneity and context-dependent polarization of DRG macrophages by conducting a focused analysis of a single-nucleus transcriptomics dataset, which was previously published for the analysis of neurons by Renthal et al. [11]. We selected this single-nucleus dataset instead of whole-cell sequencing dataset for its rare design involving several PNI models and time points, allowing us to assess the dynamic, injury-dependent changes in macrophages without the specific pre-selection of certain cell types or live cell isolation steps which could bias the recovered phenotypes of these highly plastic cells. We demonstrate the presence of unique macrophage subtypes in the mouse DRG and temporal responses to specific PNI conditions.

## 2. Materials and Methods

### 2.1. Single-Nucleus Transcriptomics

Previously published single-nucleus transcriptomics data GSE154659 were down-loaded from the Gene Expression Omnibus Database (GEO Dataset: http://www.ncbi.nlm.nih.gov/geo/) accessed on 1 June 2022. The data from DRG tissue from three PNI models, namely, spinal nerve transection (SpNT), sciatic nerve transection (ScNT), and sciatic nerve crush (Crush), and naïve controls were included from the wild-type male C57/Bl6 mice for the analysis of the macrophages. Further details on sample preparation and PNI models are provided in Renthal et al.’s original 2020 publication of the dataset [11].

### 2.2. Preprocessing and Filtering

Seurat V4 R package was used for the processing and analysis of the single-nucleus transcriptomics data [12]. To obtain high quality nuclei, only the nuclei with >600 but <50,000 total RNA counts and >500 but <10,000 RNA features were included. The nuclei with >5% abundance of mitochondrial genes were excluded.

### 2.3. Clustering and Cluster Annotation

Filtered single-nuclei data were normalized (NormalizeData), analyzed for variable features (FindVariableFeatures), scaled (ScaleData), and dimensionally reduced during the PCA (RunPCA). The first 20 principal components were used for the Nearest-neighbor graph construction (FindNeighbors), which were followed by the determination of the clusters using the resolution of 0.3 (FindClusters). Similarly, 20 dimensions were used for the visualization of clusters with Uniform Manifold Approximation and Projection (UMAP) dimensional reduction (RunUMAP). Individual samples equally contributed to the defined clusters, and thus, no further integration steps were performed.

The initial annotation of the clusters (Clustering 1) was performed based on the expression of *Rbfox3*, *Sparc,* and *Ptprc* genes to identify nuclei clusters originating from neuronal, non-neuronal, and immune cells, respectively [11]. After subsetting the immune cell-derived cluster containing *Ptprc* positive nuclei, these nuclei were re-clustered (Clustering 2 with 20 dimensions and resolution of 1.0) to define the nuclei originating from different immune cell types. The resulting clusters of immune cells were annotated based on the cell type specific marker expression profile as macrophages (*Lyz2*, *Mrc1*, *Csf1r,* and *Ccr2*), neutrophils (*S100a8*, *S100a9,* and *Retnlg*), NK/T cells *(Ms4a4b, Nkg7,* and *Cd3g*) or B cells (*Cd79a*, *Cd79b*, and *Cd19*). Finally, the nuclei belonging to macrophage clusters were selected for further analysis. The macrophages were further clustered to obtain macrophage subtypes (Clustering 3) using 0.3 as the resolution.

### 2.4. Gene Expression Scoring of Macrophage and Neuron Identities

To observe the effects of the PNI on the DRG macrophages, temporal profiles of the enrichment of injury-related genes were analyzed after the SpNT, ScNT, and Crush. The scoring for the expression of ‘Stress and inflammation’ and ‘Disease-associated macrophage’ (DAM)-related genes was calculated using Seurat’s AddModuleScore function. The DAM gene signatures have been previously described for microglia [13]. To temporally correlate the expression changes of these gene panel scores in the macrophages to the concomitant injury observed in the neuronal cells, a similar analysis of all of the cells belonging to the neuronal clusters was performed using the gene set associated with neuronal injury, as defined in the original publication of this dataset [11]. This gene list includes 524 genes consisting of neuronal injury-associated genes. All of the gene lists used for the scoring can be found in Supplementary Table S1.

### 2.5. Pseudobulk Analysis of Differentially Expressed Genes

To determine the differentially expressed genes (DEGs) between the naïve and different PNI models and time points from the pseudobulk data, the RNA counts of the macrophage nuclei from each biological replicate sample were aggregated to the summed expression using Seurat’s AggregateExpression function. The replicate samples were grouped based on the injury model and time point. The DEGs were defined by DESeq2 R package (version 1.36.0), using group information as design factor and contrasting each group with the naïve group [14]. The genes with an adjusted *p*-value of <0.05 were considered to be statistically significant DEGs. Only the transcriptomic data from the naïve group and the 24, 72, and 168 h time points were used as these were available for all of the three PNI models.

### 2.6. Pathway Analysis

The pathway analysis was performed with the DEGs or macrophage cluster marker genes using Metascape [15]. For macrophage cluster markers, the top 200 genes with an adjusted *p*-value of <0.05 were used for the Metascape analysis.

## 3. Results

### 3.1. Peripheral Nerve Injuries Time-Dependently Increase the Abundance of Macrophages in the Mouse DRG

**Figure 1:**
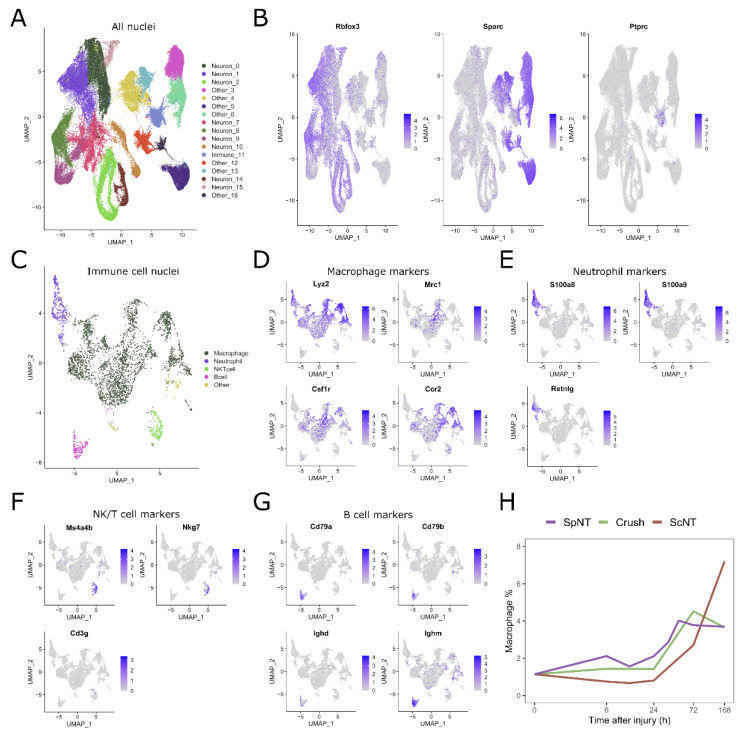
Single-nucleus transcriptomics show a proportional increase in the number of macrophages in the mouse DRG after PNI. (**A**) UMAP dimensional reduction plot showing the clustering of all cell types. Clusters 0 to 16 are annotated as neurons, immune cells, or other. (**B**) The expression of marker genes used for the annotation of neurons (*Rbfox3*), other cell types (*Sparc*) and immune cells (*Ptprc*). (**C**) Nuclei from the Immune_11 cluster were re-clustered to obtain clusters for different immune cell subtypes. (**D**) Macrophage marker genes (*Lyz2, Mrc1, Csf1r,* and *Ccr2*). (**E**) Neutrophil marker genes (*S100a8, S100a9,* and *Retnlg*). (**F**) NK/T cell marker genes (*Ms4a4b, Nkg7,* and *Cd3g*). (**G**) B cell marker genes (*Cd79a, Cd79b, Ighd,* and *Ighm*). (**H**) Percent of macrophage nuclei from the total nuclei at different time points after PNI. The scale of the x-axis is transformed to log2. The gradient color in (**B**) and (**D**–**G**) represents the level of the normalized gene expression in individual nuclei. Abbreviations: spinal nerve transection (SpNT); sciatic nerve crush (Crush); sciatic nerve transection (ScNT).

To identify the nuclei originating from the immune cells, the transcriptomics data from a naïve mouse DRG or at varying time points after different types of PNIs were retrieved from a previously published study [11] and then clustered. In total, 115,788 nuclei passed the quality control used for the filtering. The resulting 17 clusters were annotated as neuronal, non-neuronal, and immune cells (Figure 1A), while Immune_11 cluster was the only cluster expressing the immune cell marker *Ptprc* (Figure 1B).

The resulting immune cell cluster consisted of 3973 nuclei which were further clustered to identify the different immune cell types. This second, higher resolution clustering yielded 16 distinct clusters (Figure 1C). The panels of the marker genes for macrophages (Figure 1D), neutrophils (Figure 1E), NK/T cells (Figure 1F), and B cells (Figure 1G) were used to annotate these clusters and to retrieve nuclei corresponding to the macrophages for further analysis. The total number of macrophage nuclei, all of the nuclei, and the number of biological replicate samples for the naïve group and each nerve injury model time point are shown in Supplementary Table S2. Interestingly, the proportion of macrophages in the DRG was increased after all of the three PNI models compared to the naïve mice (Figure 1H).

Taken together, macrophages are present in the DRG of mice and their relative abundance is time-dependently increased upon the occurrence of the PNI.

### 3.2. The Time Course of DRG Macrophage Responses to Peripheral Nerve Injuries Is Dependent on the Site and Type of Injury

Different PNI models cause pathologies of distinct degrees. It has been shown that the temporal profile of the observed injury induction at the level of the DRG depends on the proximity of the peripheral injury [16]. Since, already, the time-dependent relative abundance of macrophages following the SpNT, ScNT, or Crush injuries was suggestive of some of the differences in the temporal profiles of macrophage responses in these models (Figure 1H), we further investigated the temporal gene expression signatures of the macrophages indicative of a response to the injury and changes of the phenotype of macrophages.

**Figure 2:**
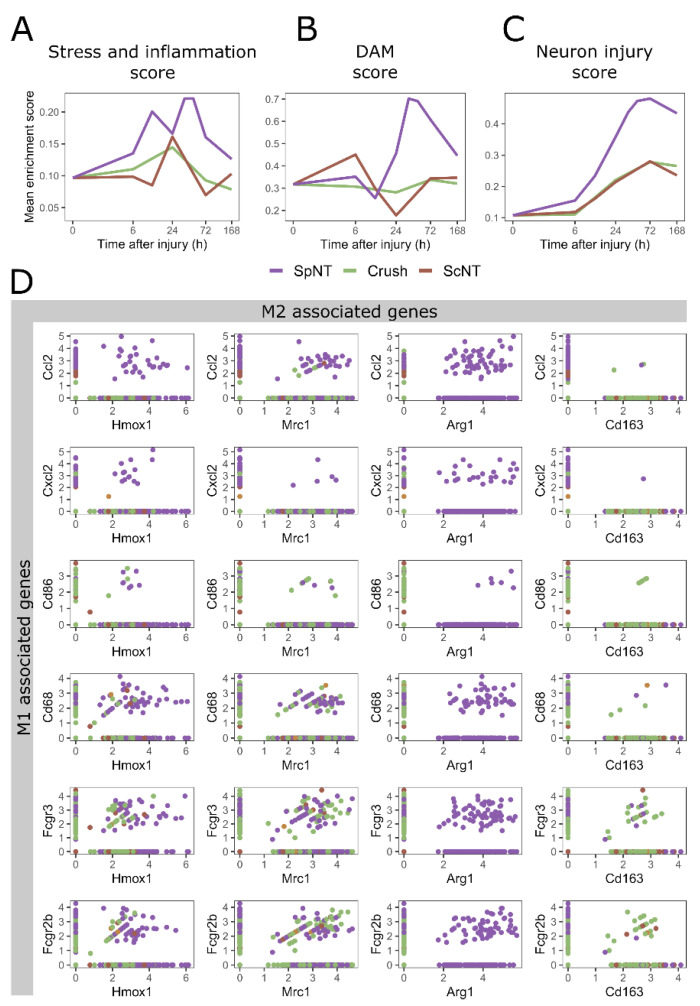
The time course of DRG macrophage responses to peripheral nerve injuries are dependent on the site and type of injury. (**A**) Mean enrichment score of stress and inflammation associated genes following different PNI models. (**B**) Mean enrichment score of DAM associated genes. All nuclei annotated as macrophages were included in the mean score in (**A**,**B**). (**C**) Mean enrichment score of neuronal injury associated genes. All nuclei annotated as neurons were included in the mean score calculation. (**D**) Co-expression of the classical M1 (y-axes) and M2 (x-axes) marker genes in individual macrophage nuclei after PNI models. Axes represent the normalized expression values. The scales of the x-axes in (**A**–**C**) are transformed to log2. Abbreviations: spinal nerve transection (SpNT); sciatic nerve crush (Crush); sciatic nerve transection (ScNT); disease-associated macrophage (DAM).

The gene expression signatures were evaluated by calculating the gene expression score for the stress and inflammation (Figure 2A) and the disease-associated macrophage (DAM) (Figure 2B)-related genes (Supplementary Table S1). Interestingly, the average stress and inflammation score of the macrophages was higher after the SpNT compared to those of the ScNT or Crush models across the analyzed time points (Figure 2A).

As for the temporal changes in the macrophage disease-associated gene expression, relatively similar DAM score levels remained across the time points analyzed for the ScNT and Crush, whilst the DAM score for the SpNT showed a temporary increase, with the highest score being observed at 36 h (Figure 2B).

To correlate the observed changes in the expression of the stress and inflammation-related genes and the DAM genes to the concomitant progression of the neuronal injury, a neuron injury score was calculated for the neuronal cells based on the expression of the injury-associated genes (Supplementary Table S1) at each analyzed time point (Figure 2C). Indeed, the neuron injury score started to increase in all of the nerve injury models during the first 24 h after the injury. As expected, based on the severity of the damage caused by the three nerve injury models [11], the SpNT induced the strongest elevation of the neuron injury score. The highest score for the SpNT increased 4.4-fold from the naïve data in comparison to the 2.5-fold increase for both the Crush and ScNT at 72 h. In respect to time, this elevation of the neuron injury score overlapped with the observed increases in the stress and inflammation score and the DAM score in the SpNT model (as seen in Figure 2A,B).

Traditionally, macrophages have been grouped into pro-inflammatory (classically activated) “M1” macrophages and anti-inflammatory (alternatively activated) “M2” macrophages. While this grouping might work to some extent in simple in vitro cell models of inflammation, it has been repetitively shown to oversimplify the nature of distinct macrophage phenotypes in vivo [4,17]. Indeed, in the studies of sciatic nerve macrophages, the mixed expression of both the M1 and M2 marker genes in the same macrophage cell was shown [2,9]. To evaluate the applicability of this simple grouping for the DRG macrophages, we explored the presence of macrophages that were positive for both the M1 and M2 genes in naïve mice or after the PNI (Figure 2D). We observed several M2 markers, such as *Arg1, Mrc1,* and *Hmox1*, to be co-expressed with M1 marker genes in the same nuclei. This was especially the case after the SpNT, underlining that the gene expression signatures of the macrophages are highly specific to the surrounding environment and to the location or type of injury.

Together, these observations suggest that the macrophages respond acutely to PNIs at the level of the DRG. The specific temporal response is dependent on the type of nerve injury, and it goes beyond traditional M1/M2 polarization.

### 3.3. Spinal Nerve Transection Modulates Inflammatory Responses of Macrophages, While Sciatic Nerve Crush Affects Their Translational Capacity

**Figure 3:**
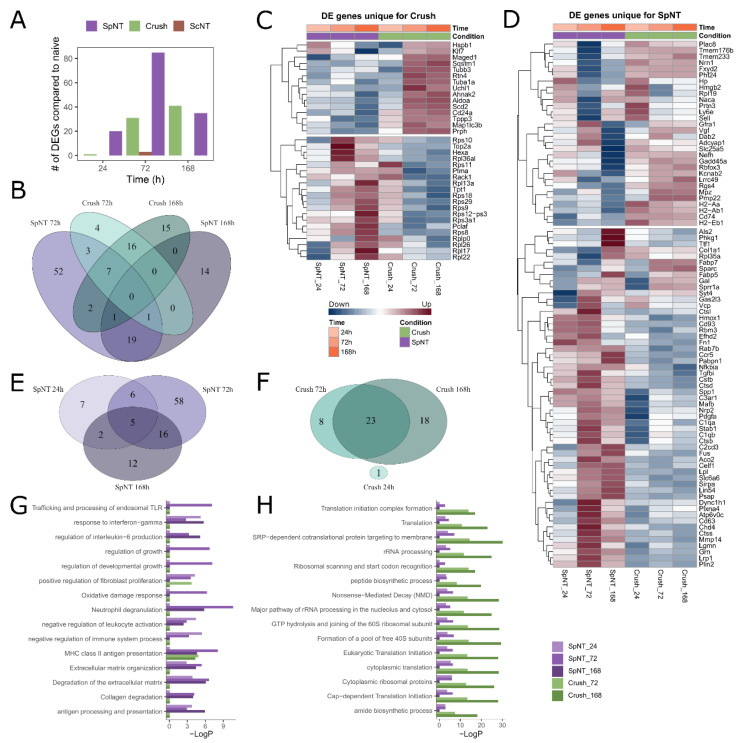
Spinal nerve transection modulates inflammatory responses of macrophages, while sciatic nerve crush affects translational capacity of macrophages. (**A**) The number of statistically significant DEGs in macrophages after different PNI models. (**B**) The number of overlapping and unique DEGs at 72 h and 168 h time points after the SpNT or Crush. (**C**) Scaled log2 fold change to naïve shown for the DEGs which were statistically significant only in the Crush model (time points 72 h and 168 h compared to the same time points of the SpNT as in (**B**)). (**D**) Scaled log2 fold change to naïve shown for the DEGs which were statistically significant only in the SpNT model. (**E**,**F**) Number of overlapping and unique DEGs at different time points in SpNT or Crush, respectively. (**G**) Fifteen most enriched pathways in the SpNT groups and their LogP values were determined using Metascape pathway analysis tool. (**H**) Fifteen most enriched pathways in the Crush groups. Abbreviations: spinal nerve transection (SpNT); sciatic nerve crush (Crush); sciatic nerve transection (ScNT); differentially expressed (DE); DE gene (DEG).

Based on the observed temporal differences in the macrophage responses (Figure 2), we analyzed the differentially expressed genes (DEGs) between the naïve and the three injury models at three common time points (24, 72, and 168 h post-injury). All of the statistically significant DEGs can be found in Supplementary Table S3. We detected fifty and one hundred and six DEGs in the Crush and SpNT time points, respectively, which were compared to the naïve one, whereas only three genes (*Ctsk, Acp5* and *Gadd45a*) reached significance for the ScNT (Figure 3A). The number of DEGs in the Crush and SpNT was higher at the 72 h and 168 h time points than they were at the 24 h time point, and thus, the number of unique and shared DEGs from these later time points (Figure 3B) and within each injury model (Figure 3E,F) were further analyzed, as shown with the Venn diagram. At 72 h, *Uchl1* was uniquely upregulated and *Pclaf, Ptma,* and *Rps12-ps3* were downregulated in Crush (Figure 3C). Several ribosomal protein mRNAs, such as *Rpl26, Rpl13a,* and *Rps10*, were uniquely downregulated, while the protein phosphorylation-related genes *Cd24a, Maged1,* and *Sqstm1* were upregulated at 168 h after the Crush. In the SpNT, several inflammation-associated genes were shown to be uniquely upregulated (*Stab1, Lrp1, Hmox1, Gal, C3ar1, Grn, C1qa,* and *Fn1*), while some of them were downregulated (*Prtn3, Sell,* and *Plac8*) (Figure 3D). At the latest SpNT time point at 168 h, the genes contributing to leukocyte activation (*Slc25a5, H2-Ab1,* and *Cd74*) were uniquely downregulated. The uniquely upregulated genes at this time point included *Col1a1, Cd2cd3,* and *Psap*.

To get an insight into the biological processes of the identified DEGs, we performed a pathway analysis with the lists of DEGs for each time point and injury model. A striking separation of the enriched pathways between the SpNT and Crush model DEGs was observed (Figure 3G,H); most of the enriched pathways for the SpNT model were involved in the inflammatory responses (Figure 3G), while for the Crush one, the most enriched pathways were related to the protein translation machinery (Figure 3H).

To support the lack of DEGs after the ScNT injury with our initial multivariate analysis approach in DESeq2 involving all of the three PNI models in the analysis design, we repeated the analysis separately for each PNI model in comparison to the naïve group. Indeed, the second analysis design did not increase the number of DEGs after the ScNT, and it showed slight differences in the detected statistically significant DEGs for each study group in comparison to the analysis in Figure 3 (Supplementary Figure S1A,B). However, the pathway analysis with the DEGs from this separate analysis of the PNI models suggests that there is an enrichment of the genes that are involved in similar biological processes as those with the original analysis (Supplementary Figure S1C).

In summary, these data suggest that there are highly specific transcriptional responses of macrophages to the SpNT and Crush injuries. The analysis of the enriched pathways suggests that these transcriptional changes likely induce functionally distinct responses related to the regulation of inflammation and the protein synthesis in the SpNT and Crush, respectively.

### 3.4. Spinal Nerve Transection Leads to the Appearance of a Particular Macrophage Cluster Associated with the Regulation of Inflammation

**Figure 4:**
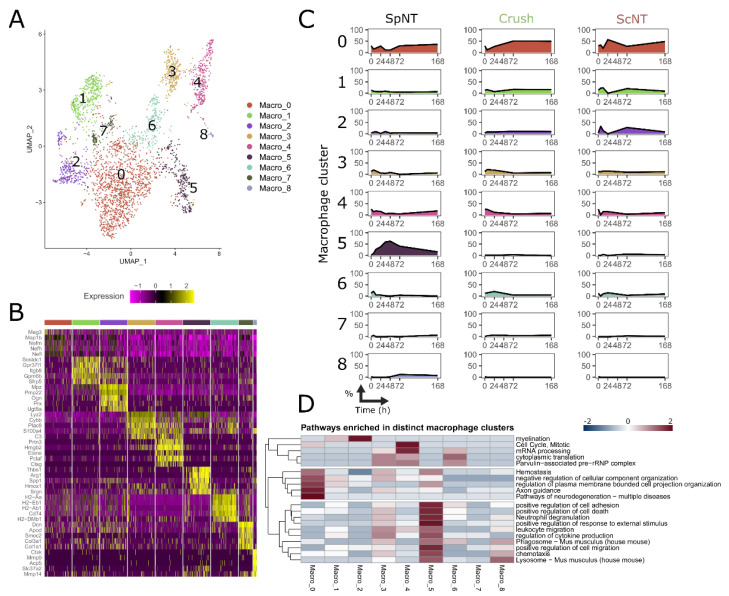
Macrophages can be divided into transcriptionally distinct clusters which correspond to varying phenotypes. (**A**) UMAP dimensional reduction plot showing the clustering of macrophages into nine distinct clusters. (**B**) Top 5 marker genes defining the macrophage clusters. (**C**) Relative proportion of each macrophage cluster shown for the different nerve injury models over time. The y-axis corresponds to the percentage of individual cluster from all macrophages and the x-axis scale is transformed to log2 for the visualization. (**D**) Summary of the most enriched pathways among the distinct macrophage clusters. The color gradient represents the LogP values calculated by the Metascape pathway analysis tool. Marker genes having adjusted *p*-value < 0.05 were used, and if over 200 genes were detected, the first 200 of them were included in the analysis. Abbreviations: spinal nerve transection (SpNT); sciatic nerve crush (Crush); sciatic nerve transection (ScNT).

We have demonstrated above the distinct responses of macrophages to the SpNT and Crush nerve injury models, suggesting that the SpNT induces a unique inflammation-related macrophage phenotype. Since several clusters were annotated as macrophages in the clustering of the immune cells (Figure 1C) and they were analyzed thus far as a one macrophage population, we posited if we could identify nerve injury-specific subpopulations of the macrophages which might explain the observed differences. To answer this question, we re-clustered the nuclei that were annotated as macrophages and defined nine different macrophage clusters (Figure 4A), each having a set of marker genes defining the cluster (Figure 4B, Supplementary Table S4). Importantly, the relative abundance of each cluster along the shared experimental time points between the injury models showed an appearance of the cluster 5 macrophages (Macro_5), specifically after the SpNT injury (Figure 4C), suggesting that the *Thbs1, Arg1, Spp1, Hmox1,* and *Srgn* expressing macrophages correspond to the spinal nerve injury-induced phenotype.

To predict the functional phenotype of the different subclusters of macrophages, we carried out a pathway analysis, with the marker genes defining these clusters (Figure 4D). Interestingly, the Macro_5 cluster showed a highly distinct enrichment of several pathways associated with cell death, chemotaxis, inflammatory responses, and phagocytosis. In addition, the analysis suggests that the clusters Macro_3, Macro_4, and Macro_6 consist of nuclei corresponding to functionally similar cells. The enriched pathways for these three clusters are associated with protein synthesis and the cell cycle process. Especially, the Macro_6 cluster contains nuclei from the Crush group, which is in agreement with the similarities of the pathway analysis with the DEGs from the Crush group in all of the macrophages (Figure 3H).

Taken together, these data suggest that the Macro_5 cluster corresponds to the nuclei from the cells that are uniquely associated with the SpNT-induced injury and have a phenotype regulating the inflammation and clearance of injured cells.

### 3.5. Nerve Injury Induces Transcriptomically Unique Macrophage Subpopulations in the DRG

To our knowledge, a detailed characterization of macrophage transcriptomics at the single-cell or -nucleus level has not been done before for DRG tissue after a PNI. The closest contexts that have been studied are the sciatic nerve macrophages after a sciatic crush [2,9] and the spinal cord macrophages and microglia after a spinal cord injury (SCI) [7,8]. Next, we compared the transcriptomics of the macrophages from our study to these earlier publications to hypothesize the functions of different DRG macrophage subpopulations after the PNI. In addition, we evaluated the heterogeneity of macrophages between different tissues.

**Figure 5:**
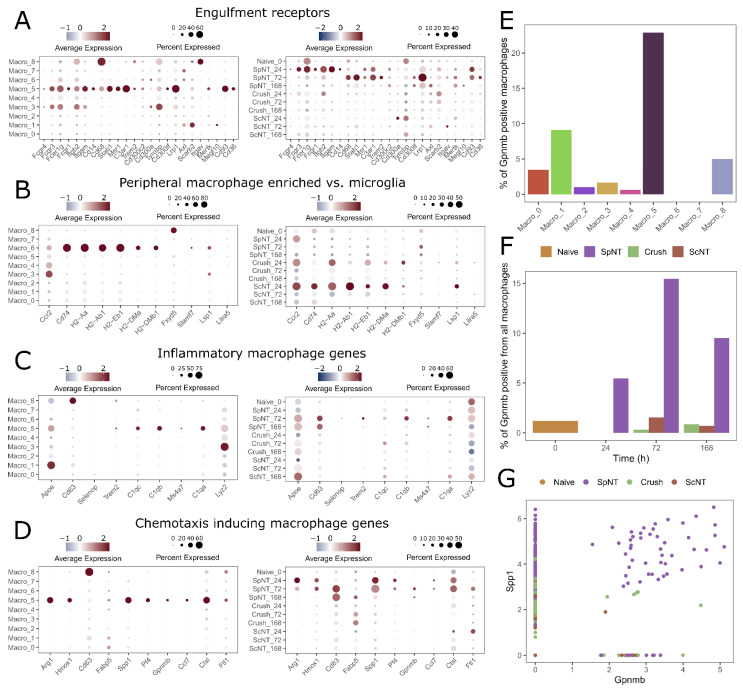
Macrophages from different tissues have distinct transcriptomic profiles and exhibit remarkable heterogeneity. (**A**) DRG macrophage expression of the phagocytosis-related engulfment receptors. Left panel shows the expression for each macrophage cluster and right panel shows the expression for each PNI model and time point. (**B**) DRG macrophage expression of the peripheral macrophage genes. (**C**) DRG macrophage expression of the inflammatory genes. (**D**) DRG macrophage expression of chemotaxis-inducing genes. (**E**) Percentage of *Gpnmb* positive DRG macrophages in each macrophage cluster. (**F**) Percentage of *Gpnmb* positive macrophages at different time points after the nerve injury models. (**G**) Co-expression of *Gpnmb* and *Spp1* following different nerve injury models. Abbreviations: spinal nerve transection (SpNT); sciatic nerve crush (Crush); sciatic nerve transection (ScNT); central nervous system (CNS); spinal cord injury (SCI).

A single-cell transcriptomic analysis from the sciatic nerve three days after the sciatic nerve crush showed the presence of five distinct macrophage clusters and one monocyte cluster [2]. These clusters were shown to express several phagocytosis-associated engulfment receptors. We visualized the expression of the engulfment receptors in the DRG macrophage clusters discovered by our analysis. The engulfment receptors were mainly expressed by the Macro_5 cluster, and the highest expression pattern corresponded to the SpNT 24 h and 72 h time points (Figure 5A). Similarly, a high expression of engulfment receptors in the DRG macrophages was not shown after the Crush, suggesting that the sciatic nerve crush induces phagocytosis by local macrophages in the peripheral nerve, but this effect is not seen further away from the injury site in the DRG macrophages.

Another recent study on sciatic nerve macrophages reported the presence of two distinct macrophage populations in the intact sciatic nerve and additional four macrophage clusters one or five days after the sciatic nerve crush [9]. Importantly, these clusters of naïve cells were found to have distinct spatial localization in the sciatic nerve, the Relmα + Mgl1+ macrophages were shown to be epineurial, and the Relmα—Mgl1—macrophages were shown to be endoneurial. We aimed at comparing the DRG macrophage clusters from our analysis to these sciatic nerve macrophages, but we found that most of the sciatic nerve macrophage cluster signature genes reported in the study [9] were expressed in only a few individual macrophages that were spread across the clusters, or they were not expressed in the dataset of our analysis. However, a set of genes that was shown to be enriched in the endoneurial sciatic nerve macrophages and not expressed by the CNS microglia formed an exception, as several of these genes (*Cd74, H2-Aa, H2-Ab1, H2-Eb1, H2-DMa,* and *H2-DMb1*) were specifically expressed in the DRG Macro_6 cluster in our analysis (Figure 5B). These genes had a higher expression level in the DRG macrophages after the Crush and ScNT injuries, especially at the 24 h time point, although their upregulation did not reach statistical significance. These genes were poorly expressed after the SpNT, and *Cd74, H2-Aa, H2-Ab1,* and *H2-Eb1* were significantly downregulated at least one time point after the SpNT when they were compared to the expression levels in the naïve mice. From these genes, *H2-Aa, H2-Ab1,* and *H2-Eb1* were among the top differentially expressed genes in the sciatic nerve macrophage group 5, corresponding mainly to the cells from the day 5 post-crush injury mice, and they were associated with the antigen presentation functions in the reference dataset [9]. This is in line with our finding in Figure 3G pathway analysis, where an enrichment of genes related to MHC class II antigen presentation in the DRG macrophages after the Crush was shown, suggesting that some similarities between the responses of the macrophages in the sciatic nerve and DRG exist after a sciatic nerve crush injury, but overall, the macrophage transcriptomics are highly specific to the tissue of residence.

To mirror the Macro_5 DRG macrophages of our study to the previous studies of macrophages in the spinal cord, we visualized the gene expression patterns described for the inflammatory and chemotaxis-inducing macrophages after the SCI [8]. We show that the DRG Macro_5 cluster has an enriched expression of the marker genes in both of the spinal cord macrophage clusters (Figure 5C–D). Injury-associated spinal cord microglia have been previously shown to uniquely express *Gpnmb* and only these cells co-expressed *Spp1* and *Igf1*, a co-expression which has been shown to promote neuroregeneration after an injury [7]. In the dataset of our study, *Igf1* is practically not expressed (data not shown). However, over 1/5 of the Macro_5 nuclei expressed *Gpnmb* (Figure 5E), and the highest fraction of *Gpnmb* positive nuclei was observed at 72 h after the SpNT (Figure 5F). Intriguingly, the co-expression of *Gpnmb* and *Spp1* was uniquely observed after the SpNT (Figure 5G). These data suggests that the SpNT-associated DRG Macro_5 cluster might be activated similarly to the spinal cord microglia upon the occurrence of the SCI. 

Taken together, the comparative analysis of the DRG macrophage clusters that was performed using the PNI models with the existing literature on the nerve injury induced macrophages suggests that partly similar transcriptional changes occur after a sciatic nerve or spinal cord injury at the sites of the injured tissues, while major differences in the tissue specific macrophages also exist.

## 4. Discussion

**Figure 6:**
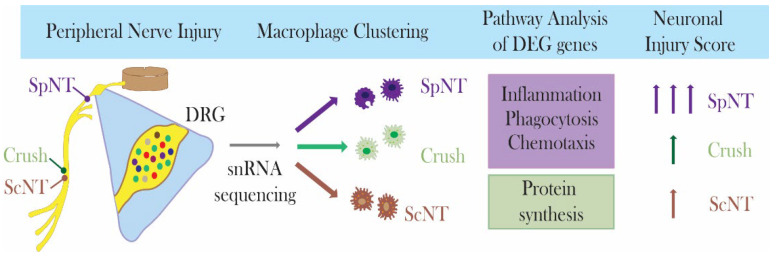
Sciatic and spinal nerve injuries show distinct effects on DRG macrophages.

Here, we have shown that DRG macrophages increase in number following the occurrence of different peripheral nerve injuries. This increase occurs concomitantly with altered transcriptomics and the induction of neuronal injury. The degree of neuronal injury was time- and injury model-dependent, being more severe after an injury affecting the spinal nerve occurred than after one affecting the sciatic nerve occurred. Likewise, the changes in the gene expression of the DRG macrophages were strongly dependent on the PNI model, suggesting that various macrophage functions are carefully orchestrated under different conditions (Figure 6).

The Crush of the sciatic nerve causes a fully reversible nerve injury by the induction of nerve regeneration, and it has been used to study nerve regeneration-promoting genes [11]. In our analysis, the macrophage nuclei derived from the Crush group had a higher temporal expression of antigen presentation-related genes such as *H2-Aa, H2-Ab1,* and *H2-Eb1* at the 24 h time point compared to those in the SpNT group after which the expression of these genes was reduced. The statistically significant downregulation of protein translation-related genes, such as *Rps12-ps3, Rpl26,* and *Rpl13a*, accounted for the major transcriptomic change from 72 h onwards after the Crush. The cells exposed to stress adapt to the changed conditions by increasing the expression of stress genes and inhibiting the expression of the other genes to reserve the capacity for recovery [18]. *Rpl13a* encodes a ribosomal protein which inhibits the translation of IFN-γ-induced inflammatory cytokines in monocytes and macrophages, leading to the suppression of pro-inflammatory responses [19]. Thus, the downregulation of *Rpl13a*, and several other downregulated ribosomal protein transcripts detected in our analysis, could influence the inflammatory phenotype of the Crush-induced macrophages. Concomitantly, the expression of *Sqstm1* and *Hspb1*, proteins associated with the stress responses and autophagy [20], respectively, were upregulated, and the cell cycle and proliferation markers *Pclaf* and *Top2a* [21] were downregulated. Interestingly, most of these genes initially showed a trend towards increased expression at the 24 h time point, suggesting highly dynamic shifting of the macrophage response. We hypothesize that these changes might reflect specific stress responses of the DRG macrophages to the crush injury of the sciatic nerve. The Macro_6 subcluster constituted a slightly higher proportion of the Crush group-derived macrophages during the first few days after the injury, and the marker genes defining this cluster were associated with protein translation and mRNA processing. The Macro_6 marker genes were also related to the antigen presentation, and they overlapped with the markers, distinguishing the peripheral sciatic nerve macrophages from the CNS microglia [9]. Thus, the Macro_6 cluster likely represents the phenotype promoted by the Crush injury.

The pathway analysis revealed that the DEGs between the SpNT and naïve macrophages regulate the inflammatory responses, such as immune cell activation, oxidative damage response, cytokine production, and the modulation of the extracellular matrix. We saw an upregulation of several inflammatory response-related genes, such as *Stab1, Lrp1, Hmox1, Gal, C3ar1, Grn, C1qa,* and *Fn1*, only after the SpNT injury, specifically at the 72 h time point. Increased antioxidative *Hmox1* expression has been shown to improve the outcome of an SCI by reducing the inflammation and oxidative stress [22], suggesting that macrophages that have an increased expression of *Hmox1* could have a protective effect instead of being injury promoting. However, the phenotype of SpNT-associated macrophages is likely more complex as the list of upregulated genes also contains complement genes, such as *C1qa*, which has been implicated in the neurodegeneration through the induction of inflammasome formation and a harmful inflammatory response in macrophages [23]. We observed the highest percentage of the Macro_5 subcluster at 48 h after the SpNT, slightly preceding the highest number of total macrophages and the maximum detected nerve injury score. However, it remains uncertain if the recruitment of Macro_5 is caused by the neuronal injury or if the degree of neuronal injury is further aggravated by the appearance of these macrophages or if it is due to both of them. Interestingly, the Macro_5 cluster was exclusively observed after the SpNT injury, and the pathway analysis suggested that there are similar functions for the Macro_5 as for the SpNT group at the 72 h time point. These inflammation-related predicted functional consequences of the altered gene expression profiles were also in line with the existing literature on macrophage phenotypes in the spinal cord [8]. In our analysis, the genes that were shown to be enriched in inflammatory macrophage and chemotaxis-inducing macrophage phenotypes after the SCI were shown to be expressed mainly by the Macro_5 cluster and after the SpNT injury. This suggests some similar functional responses between the DRG macrophages upon the occurrence of the PNI and the spinal cord macrophages after the SCI. In the spinal cord, the macrophages have been shown to play a central role in the regulation of the spinal cord microglia by limiting their inflammatory responses after a superficial nerve injury, but this mechanism was shown to fail after the spared nerve injury, which causes pathological neuropathy [24]. Another single-cell transcriptomics study on the effects of SCIs reported the presence of an injury-associated regenerative microglia subtype in the spinal cord [7]. These regenerative microglia were specifically marked by the expression of *Gpnmb* and co-expression of *Spp1*. Interestingly, the nuclei co-expressing these two genes were solely observed after the SpNT in our analysis. To hypothesize, based on our differential expression analysis, the functional prediction using pathway analysis and the comparison with the existing reports on macrophage/microglia phenotypes in the spinal cord, the Macro_5 cluster could harbor regenerative or protective functions that try to limit the nerve injury, but the capability to prevent the pathology induced by the spinal nerve injury might remain insufficient. However, more harmful functions of the DRG macrophages upon the occurrence of the PNI have also been suggested by a previous study, showing that the depletion of the DRG macrophages improves the pain behavior after the PNI [3]. Overall, the existing literature are very scarce and use variable in vivo models for studying the PNI. To our knowledge, this is the first analysis suggesting the endogenous recruitment of regenerative macrophage population in the DRG upon the occurrence of an injury to the spinal nerve.

Interestingly, our analysis did not detect large differences in the DEGs in DRG mac-rophages after the ScNT injury, despite the increase in the macrophage number. This discrepancy between Crush and ScNT, which were both performed at the sciatic nerve, may be explained by the surgical procedure and study design of the original publication. In the original publication, after the clean cutting of the sciatic nerve (ScNT), a tight ligation of the proximal end was performed to prevent regeneration, whereas for the Crush no such ligation was applied [11]. To our knowledge, this analysis provides a first indication that such a discrepancy between the effects of transection with regenerative prevention and crush on DRG macrophages might exist. However, further studies are required to confirm this hypothesis.

The polarization of macrophages did not follow traditional classification of pro-inflammatory M1 and anti-inflammatory M2 phenotype. Previous studies analyzing the in vivo phenotypes of macrophages at the single-cell level have also produced similar conclusions [2,9]. In our analysis, several *Arg1* positive macrophages, which are thought to be a marker for regenerative anti-inflammatory macrophages, also expressed pro-inflammatory genes, and this phenomenon occurred especially after the SpNT injury. This observation has far-reaching consequences regarding the traditional use of the expression of only a few anti- or pro-inflammatory genes in drawing conclusions of the macrophages’ phenotype and functions in different pathological conditions. Furthermore, the markers that are used to also identify or isolate macrophages for the downstream analysis of gene expression or function might specifically enrich the macrophages of a certain phenotype, excluding others, and this may lead to biased conclusions, as the disease condition may also modulate the expression of these genes. Our study highlights the importance of moving away from the simplistic M1/M2 labels and towards a more detailed description of the identity of the macrophages in question.

In summary, our analysis shows that different PNI models, representing distinct regenerative capacity and neuronal injury levels, induce distinct transcriptional changes in the DRG macrophages. The manipulation of the sciatic nerve by a crush or a transection with the complete prevention of the regenerative response causes remarkably distinct macrophage phenotypes at the DRG level compared to the transection of the spinal nerve. These studies suggest that while a PNI induces macrophage responses in different peripheral and central tissues, the consequences of such effects are highly dynamic, location dependent, and specific to the type of nerve injury. The goal of the studies on nerve injuries is to understand the mechanisms of injury and repair. However, our analysis is insufficient on its own for drawing conclusions on the consequences of the different DRG macrophage gene expression profiles on the recovery of injured nerves. To answer these questions, further studies using unbiased methods at the single-cell level are needed in the future. Nonetheless, the data presented here support the further investigation of the biological significance of distinct DRG macrophage subsets on neuronal functions.

## Data Availability

The data presented in this study are openly available in Gene Expression Omnibus Database at https://www.ncbi.nlm.nih.gov/geo/, reference number GSE154659 and were accessed on 1 June 2022.

## Supplementary Materials

The following supporting information can be downloaded at: https://www.mdpi.com/article/10.3390/biomedicines10123295/s1, Table S1: Gene lists for functional phenotype scoring; Table S2: Number of nuclei and replicate samples used in the analysis; Table S3: Differential gene expression analysis results for significantly differentially expressed genes; Table S4: List of all macrophage subcluster markers.
