# Supplementary material for: Re-Analysis of Single-Nucleus Transcriptomics Reveals Diverse Dorsal Root Ganglia Macrophage Responses Following Peripheral Nerve Injury
Source: Biomedicines. 2022 Dec 19;10(12):3295. doi: 10.3390/biomedicines10123295 (PMC9775330; doi:10.3390/biomedicines10123295)
Supplement: Supplementary file 1 [file biomedicines-10-03295-s001.zip › Supplementary Figure S1.pdf]

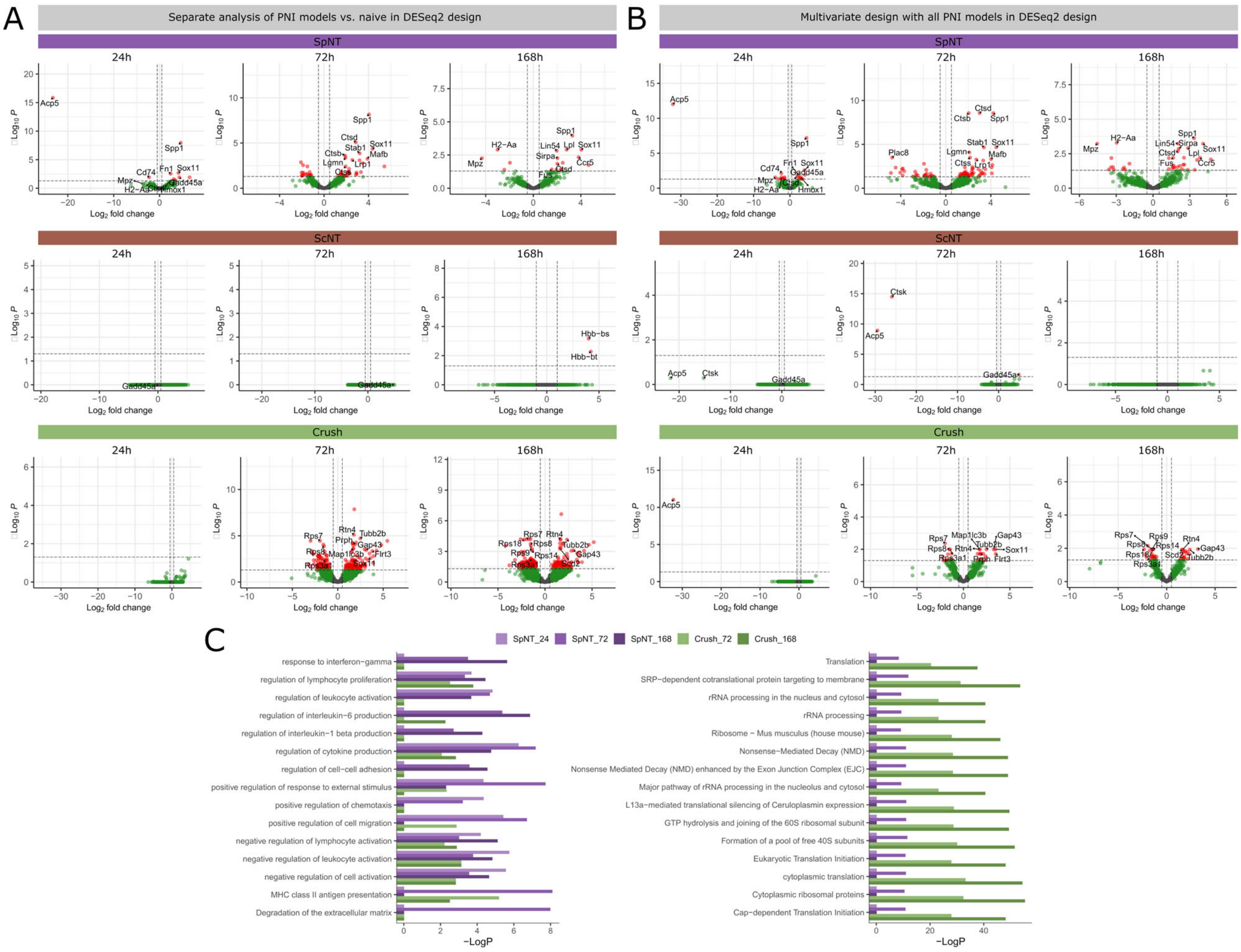

Supplementary Figure S1. Comparison of the two DESeq2 differential expression analysis approaches. (**a**) Analysis separately for each PNI model and its time points, contrasted to naïve; (**b**) Multivariate analysis design including all PNI models together, contrasted to naïve; (**c**) Pathways with highest enrichment in the SpNT (left) and Crush (right) groups using DEGs from DESeq2 analysis separately for each PNI model as in (**a**). Volcano plots in (**a**) and (**b**) show the annotation for the 10 genes with lowest adjusted p value with multivariate analysis (**b**) which was used in the analysis of the main text.
